# A case report of Barrett's esophageal adenocarcinoma in a young adult aged 20 years

**DOI:** 10.1002/deo2.70111

**Published:** 2025-04-07

**Authors:** Takayuki Ohi, Fumisato Sasaki, Nobuhisa Maeda, Shohei Uehara, Hidehito Maeda, Akihito Tanaka, Shuji Kanmura, Akihiro Yamasuji, Akio Ido

**Affiliations:** ^1^ Digestive and Lifestyle Diseases Kagoshima University Graduate School of Medical and Dental Sciences Kagoshima Japan; ^2^ Department of Gastroenterology Ikeda Hospital Kagoshima Japan

**Keywords:** Barrett's esophageal adenocarcinoma, Barrett's esophagus, esophageal neoplasms, image enhanced endoscopy, young adult

## Abstract

In Japan, the prevalence of Barrett's esophageal adenocarcinoma (BEA) has recently increased owing to a decrease in the number of patients with *Helicobacter pylori* infection, westernization of the diet, and an increase in obesity prevalence. However, BEA in patients in their 20s is extremely rare. Our patient was a 20‐year‐old Japanese woman with chief complaints of vomiting and nausea. Esophagogastroduodenoscopy was performed to investigate the cause of vomiting, and a raised lesion was found in the gastroesophageal junctional zone. In the magnified observation, the mucosal pattern of the lesion was partially invisible, and the vascular pattern was irregular; the lesion was diagnosed based on the Japan Esophageal Society classification for Barrett's esophagus ‐related superficial neoplasia. Endocytoscopic observations revealed a highly irregular glandular structure. Computed tomography showed no distant metastasis. Based on these results, we diagnosed BEA as short‐segment Barrett's esophagus and performed an endoscopic submucosal dissection. The pathological diagnosis was pT1a‐DMM. It was a well‐differentiated adenocarcinoma and was treated with curative resection.

BEA is extremely rare in young adults in their 20s. Nonetheless, appropriate surveillance is required for patients with multiple risk factors, including obesity and exposure to acid and bile resulting from persistent vomiting.

## INTRODUCTION

In Western countries, the proportion of adenocarcinomas in esophageal cancers is ≥60% and on the rise^1^ In Japan, the number of cases of Barrett's esophageal adenocarcinoma (BEA) has also increased recently due to a decrease in the number of patients with *Helicobacter pylori* infection, westernization of the diet, and an increase in obesity prevalence.^2^ BEA is more common in older men from an epidemiological perspective,^2^, ^3^ and BEA in young adults in their 20s has been rarely reported, in Japan and the West.

### Case

The patient was a 20‐year‐old Japanese woman with chief complaints of vomiting and nausea. She had a history of schizophrenia and was prescribed multiple antipsychotics. There was no family history of gastric or esophageal cancer. At a previous clinic, she underwent esophagogastroduodenoscopy to evaluate frequent vomiting, which revealed a flat, elevated lesion at the esophagogastric junction. She subsequently visited our department. The patient was obese, with a height of 158.2 cm, a weight of 83.8 kg, and a body mass index of 33.5 kg/m^2^.

**FIGURE 1:**
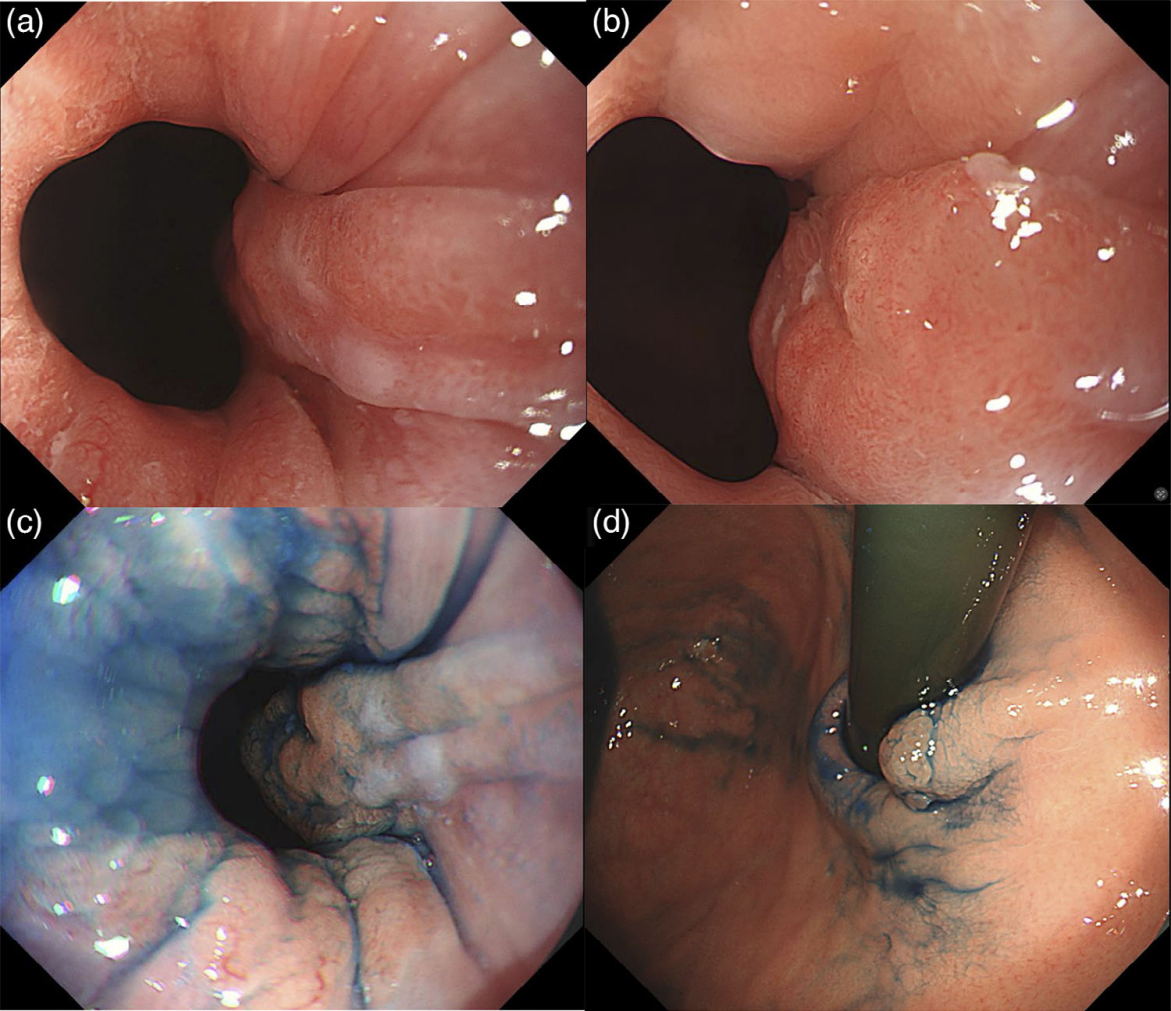
Endoscopic white light imaging pictures. (a) The flat elevated lesion is observed with a short segment of Barrett's esophagus. (b, c) The lesion is located at the 2 o'clock position, and a slight depression is observed in the center of the lesion. (d) The anal border of the lesion is clearly observed from the retroflex position.

**FIGURE 2:**
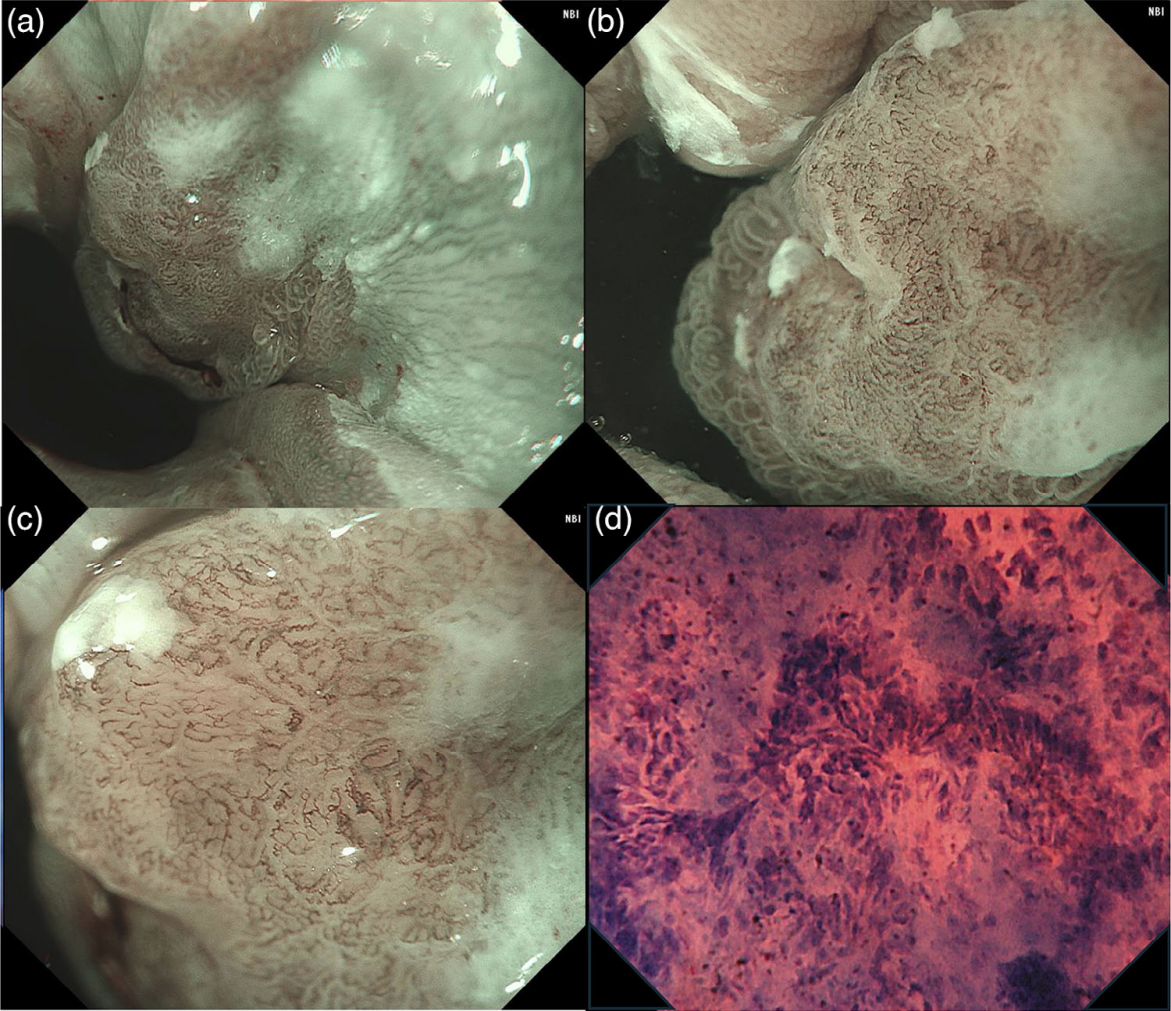
Endoscopic pictures of narrow‐band imaging and endocytoscopic imaging. (a) The lesion is observed as a brownish area. (b, c) The mucosal pattern of the lesion is partially invisible, and the vascular pattern is visible and irregular. (d) Endocytoscopic observation showed disturbed polarity of the cellular arrangement and a highly irregular glandular structure.

**FIGURE 3:**
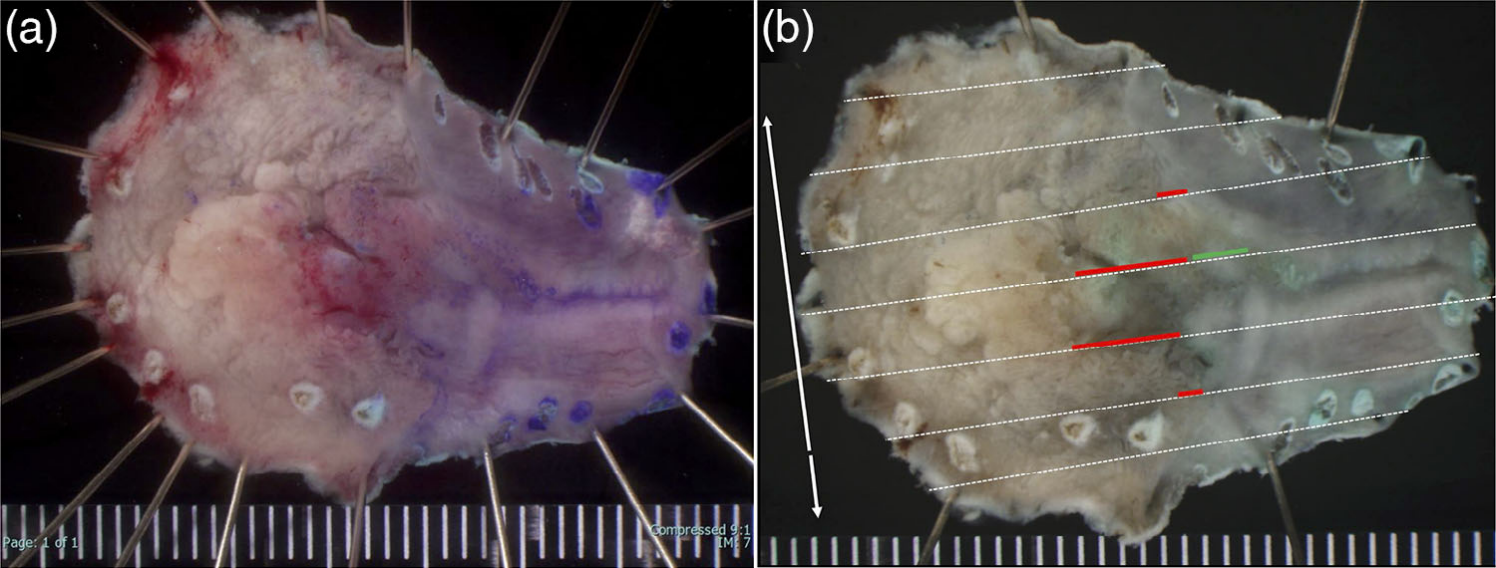
Resected specimen and mapping. (a) The tumor measures 12 × 9 mm. (b) The red line shows a well‐differentiated adenocarcinoma in the depressed area. The oral side of the lesion shows subcutaneous extension in the area indicated by the green line.

Esophagogastroduodenscopy was performed at our hospital. White light imaging revealed a flat, elevated lesion approximately 10 mm in size with an erythematous tone at the 2 o'clock position. The lesion was associated with short segment Barrett's esophagus, with a circumferential extent of metaplasia measuring 1.5 cm and a maximal extent of metaplasia measuring 2.0 cm (C1/M2). Additionally, reflux esophagitis (Los Angeles classification grade A) and a hiatal hernia were observed (Figure 1a,b). Observation using indigo carmine showed a depression in the center of the flattened elevation (Figure 1c,d). In the magnified observation with narrowband imaging, the lesion could be recognized as a brownish area (Figure 2a). We diagnosed the lesion using the Japan Esophageal Society classification for Barrett's esophagus (BE) related to superficial neoplasia.^3^ The mucosal pattern of the lesion was partially invisible, whereas the vascular pattern was visible and irregular (Figure 2b,c). Endocytoscopic observation showed disturbed polarity in the cellular arrangement within the lesion depression and a highly irregular glandular structure (Figure 2d). The lesion was easily deformed with changes in the amount of air, and endoscopic ultrasonography did not reveal submucosal invasion. No distant metastases were observed on contrast‐enhanced computed tomography. Based on these results, we diagnosed BEA (Jz, 10 mm, cType 0‐IIa+IIc, tub1, cT1a[M], cN0, cM0, and cStage IA) with a short segment BE background and performed endoscopic submucosal dissection. The resected specimen measured 32 × 19 mm, and the tumor measured 12 × 9 mm, with a depression in the center of the flat elevation. A well‐differentiated adenocarcinoma was located in the depressed area. The oral side of the lesion showed a subcutaneous extension in the indicated area (Figure 3).

**FIGURE 4:**
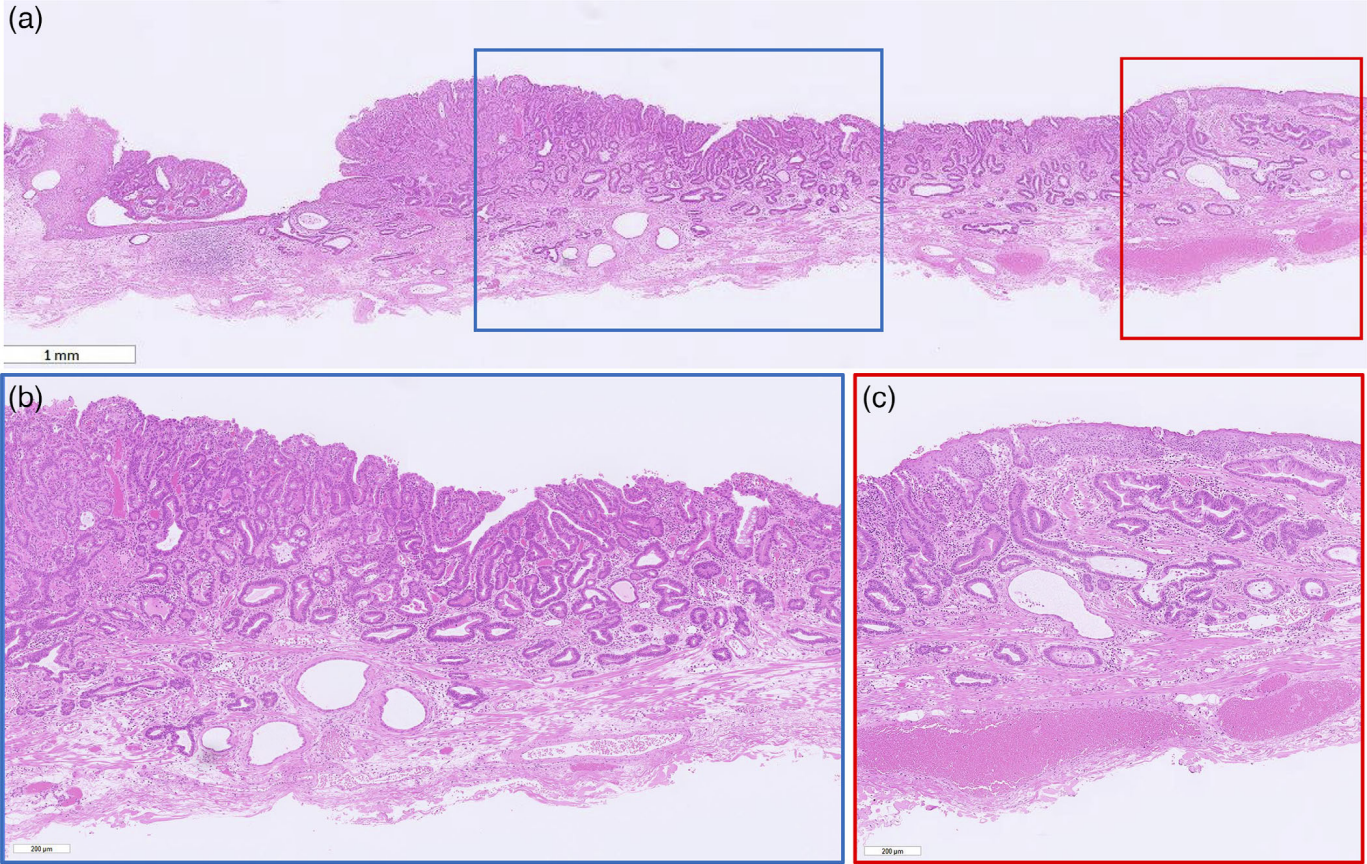
Histopathological image. (a) The background mucosa exhibits features of Barrett's esophagus, as the mucosal muscularis is multilayered and contains stratified squamous epithelium and esophageal glandular ducts. (b) The atypical glandular epithelium is proliferating, forming irregular tubular glandular structures of various sizes. (c) The carcinoma extends to the multilayered muscularis mucosae and partially shows subepithelial extension.

Histopathologically, the atypical glandular epithelium proliferated, forming irregular tubular glandular structures of various sizes. The background was considered BE, as the mucosal muscularis was multilayered and contained stratified squamous epithelium and esophageal glandular ducts (Figure 4a,b). The carcinoma extended to the multilayered muscularis mucosae and partially showed subepithelial extension (Figure 4c). Victoria Blue staining and CD31 and D2‐40 immunostaining showed no lymphovascular invasion.

## DISCUSSION

The incidence of BEA has increased rapidly in Western countries.^1^ Although squamous cell carcinoma still accounts for most cases in Japan, the proportion of adenocarcinoma cases has increased from 1.7% in 1995 to 7.4% in 2015.^4^ The total number of cases of adenocarcinomas of the gastroesophageal junction, including BEA, is also increasing, with an increasing trend in all age groups, including young people.^2^ However, the percentage of patients with BEA aged <50 years is approximately 9%, and the rate in those aged <30 years is even lower.^5^ Only one report of BEA in Japanese patients aged <30 years exists in PubMed and the Japanese Medical Abstracts.^6^ This report describes an extremely rare case involving a 20‐year‐old patient with BEA. BEA originates from BE, which is divided into long segment BE of ≥3 cm and short segment BE of <3 cm and the risk of cancer differs depending on the length of the BE.^7^ The risk of cancer increases by 1.11 to 1.39 times for every 1 cm increase in the length of the BE, and the longer the BE,^8^ the higher the risk, with the annual rate of carcinogenesis of long segment BE being 1.2%.^9^ According to the 2022 edition of the Japan Esophageal Society Guidelines, esophagogastroduodenoscopy surveillance states that no recommendations can be currently made for BE with a maximum length of <3 cm.^8^ Furthermore, most guidelines recommend endoscopy for screening purposes only in patients aged ≥50 years with multiple risk factors.^2^ For this reason, BEA develops in young patients at a more advanced stage, and they have a poorer prognosis than older patients.^10^ Therefore, it is essential to identify risk factors and consider early screening tests, particularly for patients with symptoms such as vomiting and reflux esophagitis.

To date, the reported clinical risk factors for BEA are congenital factors such as race and sex; acquired factors such as smoking, obesity, and diet; and other gastrointestinal‐related factors such as gastric acid, bile acid reflux, failure of the reflux prevention mechanism, nitrate in saliva, length of the BE, *H. pylori* infection, and hiatal hernia.^3^ In addition, factors that contribute to the onset of the disease, particularly in young people, are obesity (body mass index ≥30 kg/m^2^) and gastric acid and bile acid reflux.^2^ This patient was considered to have a high risk of developing BEA owing to multiple risk factors, including obesity, exposure to acid and bile due to persistent vomiting, and hiatal hernia. There was no family history in this case, suggesting a low likelihood of genetic factors. Although previously reported cases were not obese, they exhibited symptoms of gastric acid and bile acid reflux.^6^

As the number of cases of BEA in Japan increases, there is a possibility of increasing the number of cases among young people. Therefore, as in the present case, it is necessary to conduct appropriate surveillance of patients with multiple risk factors.

## CONFLICT OF INTEREST STATEMENT

None.

## ETHICS STATEMENT

All procedures were performed in accordance with the ethical standards of the Declaration of Helsinki and its later amendments.

## PATIENT CONSENT STATEMENT

Informed consent was obtained from the patient for the publication of this case report.
